# Tegsedi (Inotersen): An Antisense Oligonucleotide Approved for the Treatment of Adult Patients with Hereditary Transthyretin Amyloidosis

**DOI:** 10.3390/ph12020078

**Published:** 2019-05-21

**Authors:** Luís Gales

**Affiliations:** 1 i3S—Instituto de Investigação e Inovação em Saúde, Rua Alfredo Allen, 208 Porto, Portugal; lgales@ibmc.up.pt; Tel.: +351-22-040-8800; 2 IBMC—Instituto de Biologia Molecular e Celular Universidade do Porto, Rua Alfredo Allen, 208 Porto, Portugal; 3 ICBAS—Instituto de Ciências Biomédicas Abel Salazar, Rua de Jorge Viterbo Ferreira 228 Porto, Portugal

**Keywords:** Tegsedi (Inotersen), gene silencing, antisense oligonucleotide, transthyretin, hereditary transthyretin-mediated amyloidosis (hATTR), hATTR treatment

## Abstract

Tegsedi (Inotersen) is a chemically modified antisense oligonucleotide that inhibits the hepatic production of transthyretin (TTR). Several single-point mutations in TTR destabilize its structure, leading to the aggregation and accumulation of amyloid deposits in the nervous system, heart, kidneys and eyes. In July 2018, Tegsedi was approved by the European Commission for use in adults with stage one and two polyneuropathies. Later on, in October 2018, the FDA and Health Canada also approved its use for the treatment of the polyneuropathy of hereditary transthyretin-mediated amyloidosis (hATTR) in adults in the U.S. and Canada. Tegsedi was developed by Ionis Pharmaceuticals, the company that holds the global marketing license, together with its subsidiary Akcea Therapeutics.

## 1. Introduction

Transthyretin (TTR) is one of the three major thyroid hormone-binding proteins in human plasma. It is a homotetramer, with the monomers assembled around a central channel that can accommodate two thyroxine molecules [1]. TTR may also form a complex with retinol-binding protein (RBP), being thus also associated with the transport of retinol. The hereditary form of TTR amyloidosis is an autosomal dominant disease caused by single-point mutations in the TTR gene that originate TTR variants with reduced stability prone to dissociation into non-native monomers, which in turn rapidly self-assemble into oligomers and ultimately amyloid fibrils that accumulate in the nervous system, heart, kidneys and eyes. There are multiple TTR variants, the most frequent being TTR V30M [2], the first identified cause of Familial Amyloidotic Polyneuropathy. Common clinical manifestations of TTR amyloidosis include peripheral neuropathy, cardiomyopathy, autonomic dysfunction, diarrhea and constipation. The non-hereditary form of the disease is mainly associated with the deposition of amyloid aggregates of wild-type TTR in the hearts of older people. The disease’s main areas of focus are located in Portugal, Sweden, Japan, Brazil, Italy, France, and USA.

The main treatment option for hATTR patients is a liver transplant (20-year survival rate of 55.3% after treatment [3]). This therapeutic approach is based on the removal of the main source of the unstable mutated TTR (there is also local production of TTR in the eye by retinal pigment epithelial cells and by the choroid plexus epithelium). However, it does not prevent the progression of cardiac disease since the wild-type TTR may continue to produce amyloid deposits in the heart.

An emerging treatment strategy consists of the inhibition of hepatic production of TTR through gene silencing using small interfering RNAs [4] or antisense oligonucleotides [5]. An siRNA molecule Onpattro™ (patisaran, Alnylam) has been approved by the FDA for the treatment of hATTR amyloidosis in adults [6,7]. Antisense oligonucleotides, such as Tegsedi, are defined as chemically synthesized oligonucleotides, generally 12–30 nucleotides in length, that are designed to bind to RNA by Watson–Crick base pairing rules [8]. They are capable of specifically binding to only one target RNA and promoting its degradation or steric blockage. The chemical modification of the oligonucleotides is usually necessary to reduce their degradation rate in vivo. Six antisense drugs have received market authorization, and at least four drugs are in phase III clinical trials or submitted for market authorization [9]. The goal, in the case of Tegsedi, is to bind to TTR-messenger RNA (mRNA) and reduce the concentration of circulating TTR [10]. Tegsedi targets the TTR RNA transcript and reduces the levels of the TTR transcript through an RNaseH1 mechanism of action, leading to reductions in both mutant and wild-type TTR protein and thus potentially affecting the transport of thyroxine (though there are two other plasma protein carriers of thyroxine) and of retinol (vitamin A). Patients under Tegsedi treatment receive vitamin A supplements. It is possible to perform allele-specific knockdown of only the mutant allele but that is not the strategy followed, probably due to the multiple TTR mutations that may occur and to the fact that the wild-type protein may also be involved in TTR amyloid cardiomyopathy.

An alternative treatment strategy is to bind compounds that stabilize the native fold of TTR, inhibiting its dissociation into monomers [11,12]. The formation of non-native monomers is the key step in TTR amyloidosis. The targeted TTR binding site is usually the thyroxine binding channel (most TTR protein circulates free of thyroxine in the plasma) because the channel is formed around the weakest dimer–dimer interaction and the ligands are designed to prevent dimer dissociation. One of these compounds, tafamidis (Vyndaqel) [13], has been approved for the treatment of stage I adult patients in Europe (it was accepted for FDA Review in April 2019). Tafamidis was shown to have a positive effect in transthyretin amyloid cardiomyopathy patients [14], lowering mortality and reducing patients’ decline in functional capacity and quality of life (results from a double-blind, placebo-controlled, phase 3 trial, with 441 patients). Transthyretin amyloid cardiomyopathy is caused by the deposition of wild-type (liver transplant not useful in this case) or variant transthyretin amyloid fibrils in the myocardium. Other kinetic stabilizer candidates include natural products, such as curcumin [15] and xanthones [16], FDA-approved molecules, such as diflunisal [17] and tolcapone [18], or their iodo-derivatives (the TTR channel has three pairs of halogen binding pockets to accommodate the iodine atoms of the natural ligand thyroxine) [19,20,21]. A small number of drug candidates target the surface of TTR preventing self-association [22,23,24].

**Figure 1:**
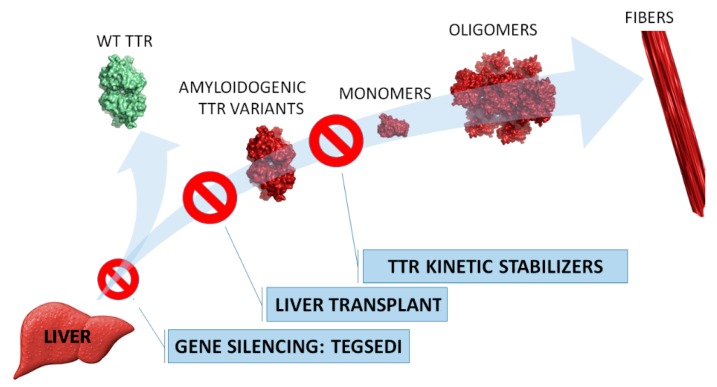
Strategies to prevent transthyretin (TTR) amyloidosis. Liver transplant is the standard practice since TTR (an amyloidogenic variant in familial amyloid polyneuropathy (FAP) patients) is mainly produced in the liver. Tegsedi inhibits hepatic production of TTR (both wild-type and amyloidogenic variants). TTR kinetic stabilizers are compounds that selectively bind to TTR, preventing its dissociation (Tafamidis was approved in E.U.)**.**

In Figure 1, the targeted intervention points in the TTR amyloidosis cascade of the abovementioned treatment strategies are highlighted.

## 2. Tegsedi (Inotersen)

### 2.1. Name

Tegsedi, also known as Inotersen, IONIS-TTR_RX_ or ISIS 420915, is a 2′-O-methoxyethyl-modified antisense oligonucleotide inhibitor of the hepatic production of transthyretin protein. It was developed by Ionis Pharmaceuticals which holds the marketing license together with its subsidiary Akcea Therapeutics. 

### 2.2. Uses

Tegsedi was approved by the European Commission in July 2018 and by the FDA and Health Canada in October 2018 for treatment in adults with stage one and two familial amyloid polyneuropathy (FAP). It should be taken once a week through subcutaneous injection of 284–300 mg. Patients should be closely monitored for renal function and platelet count.

### 2.3. Mechanism of Action

Tegsedi is a 2′-O-methoxyethyl-modified RNA molecule that binds to TTR mRNA and is designed to degrade the transcripts via the RNAseH pathway, thereby halting TTR protein production. Tegsedi does not differentiate between normal and mutated TTR mRNA, and thus the treatment reduces the circulating concentration of both the wild-type and amyloidogenic forms of the protein. In the particular case of transthyretin amyloid cardiomyopathy patients, in which the deposition of wild-type TTR in the heart is observed, the emergent strategies (gene silencing and TTR kinetic stabilization) are particularly promising as liver transplant is not effective because it does not reduce the plasma concentration of wild-type TTR. 

Tegsedi is a gapmer design with five 2′-MOE nucleotides on the 5′- and 3′-ends of the oligonucleotide, and ten DNA nucleotides in the middle to support the RNase H1 mechanism [8,10]. It was shown that Tegsedi produced a dose-dependent reduction of TTR mRNA and of the protein in culture cells and in transgenic mice. A 90% reduction in TTR RNA expression in the liver and an 80% reduction in circulating TTR protein was observed in monkeys administered with Tegsedi [10].

A single- and multiple-dose phase 1 clinical study was conducted in healthy volunteers [10]. In the multiple-dose study, Tegsedi was administered subcutaneously on days 1, 3, 5, 8, 15, and 22 in doses of 50, 100, 200, 300, or 400 mg. The mean percent reduction in plasma TTR ranged from 8% (50 mg dose group) to 76% (300 and 400 mg dose groups, no observable differences between the two groups). No serious adverse effects occurred in the study and the drug effects lasted for more than 30 days after the last administration. Based on these promising results, a double-blind phase 3 trial was initiated (see next section). 

### 2.4. Clinical Studies

A phase 3 trial (NCT01737398) of Tegsedi in adults with stage 1 or stage 2 hereditary TTR amyloidosis with polyneuropathy was run with a total of 172 patients (112 in the Tegsedi group and 60 in the placebo group), nearly half of them carrying the Val30Met mutation. Patients received weekly subcutaneous injections of Tegsedi (300 mg) or placebo and a total of 139 (81%) completed the 66 weeks of intervention. From the 33 patients that did not finish the trial, 25 belonged to the Tegsedi group and their reasons for stopping were as follows: adverse events (16), disease progression (2), meeting renal stopping rule (2), voluntary withdrawal (2), withdrawal by sponsor (2), and undergoing liver transplantation (1) [5]. The adverse events were nausea, pyrexia, chills, vomiting, anemia, thrombocytopenia, and lowered platelet counts. There were five deaths during the study, all in the Tegsedi group, four of which were consistent with the progression of the disease. One patient from the Tegsedi group had a fatal intracranial haemorrhage associated with grade 4 thrombocytopenia and another two showed severe platelet declines to below 25 × 10^3^ platelets/μL. Drug discontinuation and treatment with glucocorticoids brought platelet count to normal values. Consequently, the sponsor instituted weekly platelet monitoring, with no additional cases detected. The sponsor (Ionis Pharmaceuticals) found the effect to be specific to Tegsedi (not observed in other clinical trials of antisense oligonucleotides), which is intriguing given the fact that transthyretin has no known function in platelets and is not known to interact with other clotting factors [25].

All the patients received vitamin A supplements at the recommended daily allowance to ensure the adequate delivery of vitamin A to tissues (as mentioned in the introduction, transthyretin is involved in the transportation of vitamin A, which may be partially compromised by the reduction in transthyretin levels in the plasma). 

The primary end points were the changes, from baseline to week 66, in the standardized modified Neuropathy Impairment Score + 7 (mNIS + 7) [26,27] and Norfolk Quality of Life–Diabetic Neuropathy (QOL-DN) [28]. mNIS + 7 score ranges from −22.3 to 346.3 (the higher the score, the poorer the function), and its minimal clinically meaningful change is accepted to be 2 points [27]. The QOL-DN questionnaire ranges from −4 to 136, with higher scores indicating a poorer quality of life. Thus, for both scores, a decrease is associated with an improvement in the patient’s state. The difference in the least-squares mean change, from baseline to week 66, between the two groups (Tegsedi minus placebo) was −19.7 points (95% confidence interval [CI], −26.4 to −13.0; P < 0.001) for the mNIS + 7 and −11.7 points (95% CI, −18.3 to −5.1; P < 0.001) for the Norfolk QOL-DN score, which demonstrates the improvements reached with Tegsedi. 

Results revealed that Tegsedi slowed the progression of nerve cell damage and improved the quality of life of the patients, independently of disease stage, mutation type, or the presence of cardiomyopathy. The reduction in circulating TTR in the Tegsedi group reached around 75% by week 13 (steady-state levels). The principal concerns were kidney disease (glomerulonephritis, three patients) and low platelet counts (as mentioned above, thrombocytopenia, three patients), which were managed with enhanced monitoring.

A Phase 2 clinical trial (NCT03702829) is currently underway: 24-Month Open Label Study of the Tolerability and Efficacy of Tegsedi in TTR Amyloid Cardiomyopathy Patients. It involves 50 patients with cardiac amyloidosis due to wild-type or mutant TTR. Disease progression is measured by serial cardiac biomarkers, 6-minute walks, cardiopulmonary stress testing, advanced “strain” echocardiography and cardiac MRI. Vitamin A level (as TTR is a transport protein for Vitamin A) and TTR level are drawn to determine the degree of suppression. Every 2 weeks, blood is monitored for renal function and platelet count.

An ongoing Phase 3 trial (NCT02175004) with 135 participants is also testing the long-term (five-year) safety and efficacy of Tegsedi in FAP patients (estimated study completion date: September 2022). 

## 3. Perspectives

Tegsedi significantly reduces the concentration of circulating TTR, which ultimately improves the course of neurologic disease and the quality of life in patients. However, as stated in the FDA warning section, it causes reductions in platelet count, which may result in sudden and unpredictable thrombocytopenia, and it can also cause glomerulonephritis, which may require immunosuppressive treatment and may result in dialysis-dependent renal failure.

Very recently, Patisaran, Tegsedi and Tafamidis, were approved for the treatment of adult patients with familial amyloid polyneuropathy (FAP). The long-term evaluation of the compounds is still ongoing. Nevertheless, it is conceivable that a synergistic effect can be achieved by the combination of therapeutic molecules that target distinct points of the TTR amyloidosis cascade.
